# A Case of Superwarfarin Poisoning Due to Repetitive Occupational Dermal Rodenticide Exposure in a Worker

**DOI:** 10.4274/tjh.2015.0433

**Published:** 2016-08-19

**Authors:** Zehra Narlı Özdemir, Uğur Şahin, Mustafa Merter, Mehmet Gündüz, Berna Ateşağaoğlu, Meral Beksaç

**Affiliations:** 1 Ankara University Faculty of Medicine, Department of Hematology, Ankara, Turkey

**Keywords:** Superwarfarin, Acquired coagulopathies, Vitamin K

## Abstract

Superwarfarin poisoning is usually due to chronic occult small-dose exposures and can easily be misdiagnosed and may lead to serious complications. The diagnosis can be confirmed by a concordant history and analyses of blood and urine specimens with the liquid chromatography with tandem mass spectrometry (LC-MS/MS) technique. Several months of continuous treatment with high doses of daily oral vitamin K, as well as other supportive measures, are warranted, especially when repeated laboratory measurements to help predict the treatment period are not available. In this paper, a case of superwarfarin poisoning due to chronic repetitive occupational dermal exposure to commercial rodenticides is presented.

## Abstract (tr)

Süpervarfarin zehirlenmesi genellikle, fark edilmeyen küçük dozlarda kronik maruziyete bağlı olarak gelişir ve kolaylıkla yanlış tanı konarak ciddi komplikasyonlara yol açabilir. Tanı, uyumlu bir hikaye varlığında kan ve idrar örneklerinin sıvı kromatografisi ve tandem kitle spektrometresi (LC-MS/MS) tekniği ile analiz edilmesi yoluyla doğrulanabilir. Özellikle de, tedavi süresine karar verilebilmesi için tekrarlanan laboratuvar ölçümlerinin yapılmasının mümkün olmadığı durumlarda, aylar boyunca yüksek dozda günlük oral K vitamini verilmesi gereklidir. Bu yazıda, ticari rodentisitlerle tekrarlayan mesleksel deri maruziyetine bağlı olarak süpervarfarin zehirlenmesi gelişen bir olgu sunulmaktadır.

## INTRODUCTION

A 40-year-old man without any prior disease was referred to our outpatient clinics with a one-month history of recurrent epistaxis, ecchymoses, and hemarthroses. Administration of fresh frozen plasma (FFP) in the emergency room corrected the markedly abnormal international normalized ratio (INR), prothrombin time (PT), and activated partial thromboplastin time (aPTT) temporarily. His past medical and family histories were unremarkable. There were no bleeding episodes following surgery or trauma.

Laboratory analyses repeatedly revealed a sustained deficiency of vitamin K-dependent clotting factors with the following results: factor II, 26.9%; factor VII, 0.1%; factor IX, 13.3%; factor X, 23.5%; factor V, 82%; factor VIII, 131%; INR, 4.04; PT, 48.5 s; aPTT, 48.3 s; D-dimer, 21 ng/mL; fibrinogen, 5.01 g/L. PT and aPTT normalized in the dilution assay, excluding any acquired inhibitors. He had a hypochromic microcytic anemia concordant with chronic blood loss. The platelet count was 164x109/L. The absence of schistocytes in the peripheral blood smear excluded disseminated intravascular coagulation. Liver function tests and hepatobiliary ultrasound were normal. Endoscopic examinations of the gastrointestinal tract revealed normal mucosa. After excluding all other possible causes, intoxication with superwarfarin remained the diagnosis of exclusion. Blood and urine specimens were analyzed at the biochemistry laboratory of the Forensic Medicine Institute in Ankara and revealed a high blood level of superwarfarin, 32 ng/dL, and a positive urine test. Liquid chromatography with tandem mass spectrometry (LC-MS/MS), which allows for the identification, characterization, and quantification of chemical compounds in a specimen based on their molecular masses and fragmentation patterns [1], was used in analyses. However, various special methods do exist for the simultaneous analysis of multiple hydroxycoumarin rodenticides in a specimen [2]. Our laboratory could quantify the blood level of superwarfarin, but could give only a qualitative result for the urine specimens. We were not able to identify the specific superwarfarin compound in the blood samples, as this requires some special techniques and expertise.

Thereafter, the patient, who had been working in a car-washing facility, admitted that he was exposed to various brands of commercial rodenticides in this facility and did not take the necessary precautions (i.e. gloves and masks) while handling and scattering the rodenticidal pellets and pastes, in contrast to his coworkers, none of whom had similar symptoms. As previously reported, long-term repetitive exposure to rodenticides via direct skin contact was the probable route of systemic absorption in this case [3].

Superwarfarins are lipid-soluble long-acting coumarin derivatives available since the 1970s as strong rodenticides and they cause prolonged anticoagulation both in rats and human [4]. The second-generation anticoagulant rodenticides including brodifacoum, bromadiolone, difenacoum, difethialone, and flocoumafen have longer elimination half-lives and greater accumulation and persistence due to a greater affinity to binding sites in the liver [5]. Among these compounds, brodifacoum has been reported to have the longest plasma and liver elimination half-lives, which are 91.7 days and 307.4 days, respectively [5].

The common commercial rodenticidal formulations marketed in Turkey are in pellet and paste forms and contain 0.005% (w/w) brodifacoum, 0.005% (w/w) difenacoum, or 0.0025% (w/w) difethialone. Given the extremely dilute concentrations of these compounds in the commercial formulations, only a huge amount of a single-dose exposure may cause important side effects. In concordance, fatalities from a single-dose exposure are reported to be very rare [2]. However, regarding the long elimination half-lives and lipid solubility of these compounds, repetitive exposures to small doses of these formulations may result in chronic poisoning.

Our patient had intermittent episodes of bleeding together with prolonged PT approximately every 3-4 weeks after the outpatient clinic visit during a 6-month period. In every visit he was prescribed FFP and 10 mg of parenteral phytomenadione, and he continued taking oral phytomenadione of 10 mg daily for 7-10 days. At the end of six months his coagulation tests normalized and bleeding episodes disappeared.

The half-life of superwarfarin can be estimated by repeated measurements of blood levels in order to predict the treatment period [6]. In our case, the Forensic Medicine Institute laboratory was the only available place able to perform these kinds of analyses in the area. However, as it is subject to governmental regulations, we could not get permission for repeated measurements. Due to the presence of chronic exposure to more than one product and failure to identify the specific superwarfarin molecule with laboratory tests, it was hard to predict the outcome in our case. However, considering the long wash-out period of our case, we can speculate that the patient might have had more exposure to a product containing the superwarfarin with the longest half-life, which is brodifacoum. As we could not diagnose the patient earlier, he was given vitamin K supplementation at lower doses and shorter periods than recommended. Supplemental vitamin K doses of up to 100 mg daily for 12 months have been reported [7]. Our patient was a nonobese male, weighing 72 kg and 175 cm in height. In obese patients, considering the lipid solubility of these compounds, longer periods and higher doses may be required.

Superwarfarin poisoning due to accidental, occupational, and criminal exposures has been reported and should be suspected when an acquired bleeding disorder due to persistent deficiency of vitamin K-dependent clotting factors and a lack of sustained response to treatment with conventional doses of vitamin K are present [8]. Rodent infestation and food contamination are important sources of superwarfarin poisoning in poor socioeconomic settings. Long-term vitamin K supplementation is the recommended treatment [6]. Intravenous administration of vitamin K is often necessary initially. However, the risk of anaphylactic reactions should be considered [9].

This paper aims to refresh awareness about this clinical scenario that can easily be misdiagnosed and may lead to serious complications. Chronic occult small-dose exposures might be overlooked and should be meticulously investigated. The diagnosis can be confirmed by a concordant history and analyses of blood and urine specimens with the LC-MS/MS technique in the absence of warfarin therapy. Several months of continuous treatment with high doses of daily oral vitamin K, as well as other supportive measures, are warranted, especially when repeated laboratory measurements to help predict the treatment period are not available.

## Ethics

Ethics Committee Approval: Not applicable; Informed Consent: It was taken.
